# Successful management of retinal metastasis from renal cancer with everolimus in a monophthalmic patient: a case report

**DOI:** 10.1186/s13256-017-1501-2

**Published:** 2017-12-07

**Authors:** Ismail Essadi, Issam Lalya, Mohamed Kriet, Abdelhamid El Omrani, Rhizlane Belbaraka, Mouna Khouchani

**Affiliations:** 1 Medical Oncology, Ibn Sina Military Hospital, Cadi Ayad University, Marrakesh, Morocco; 2 Radiation Oncology, Mohamed V Military Hospital, Rabat, Morocco; 3 Cadi Ayad University, Marrakesh, Morocco; 4 Ophthalmology, Ibn Sina Military Hospital, Cadi Ayad University, Marrakesh, Morocco; 5 Radiation Oncology, Mohamed VI University Hospital, Cadi Ayad University, Marrakesh, Morocco; 6 Medical Oncology, Mohamed VI University Hospital, Cadi Ayad University, Marrakesh, Morocco

**Keywords:** Retina, Metastasis, Clear cell carcinoma, Kidney

## Abstract

**Background:**

The retina is an uncommon site for metastases, in particular from solid tumors. Some authors have reported a recent increase in the incidence of metastases in infrequent sites, such as brain or bone, probably due to the expanded treatment options and the resulting improved survival. Choroidal metastasis is the most common type of intraocular malignancy. The most common primary sites associated with choroidal metastasis are breast cancer in women and lung cancer in men. Treatment options are limited, but they must be discussed and adapted to the patient profile.

**Cases presentation:**

We report a case of a 62-year-old Moroccan man with a history of monophthalmitis secondary to a war injury of 30 years’ duration. He has been followed for 28 months for metastatic clear-cell renal carcinoma. The first-line treatment was effective for 24 months, before disease progression as retinal metastasis and accentuation of lung metastases. A second-line treatment with everolimus resulted in marked improvement of symptoms, complete recovery of visual function, and partial response in retinal localization.

**Conclusions:**

Choroidal metastasis of renal cancer is a rare situation that must be actively sought in order to arrive at a suitable therapeutic approach.

## Background

The occurrence of retinal metastasis is a rare presenting manifestation of solid tumors [1]. Metastatic tumors are the most common intraocular malignancy in adults [1]. They are most commonly found in the choroid and less frequently in the iris and ciliary body [2]. Owing to its high vascularity, the posterior choroid is particularly exposed to blood-borne cancer cells [3]. Although symptomatic choroidal metastasis is less common than asymptomatic choroidal metastasis, visual disturbance caused by cancer metastasis from other organs is one of the most important limits on the quality of life of patients with cancer [4].

Therefore, further improvement of the therapy for visual disturbance is needed. Clinically available therapies for choroidal metastasis are currently very limited [5]. Chemotherapy causes systemic adverse effects and is not always effective. Radiotherapy provides local therapy but has several complications, including cataract, exposure keratopathy, iris neovascularization, radiation retinopathy, and radiation papillopathy [5, 6]. We report a case of a patient successfully managed with everolimus as second-line treatment of retinal metastasis from renal cancer.

## Case presentation

**Fig. 1:**
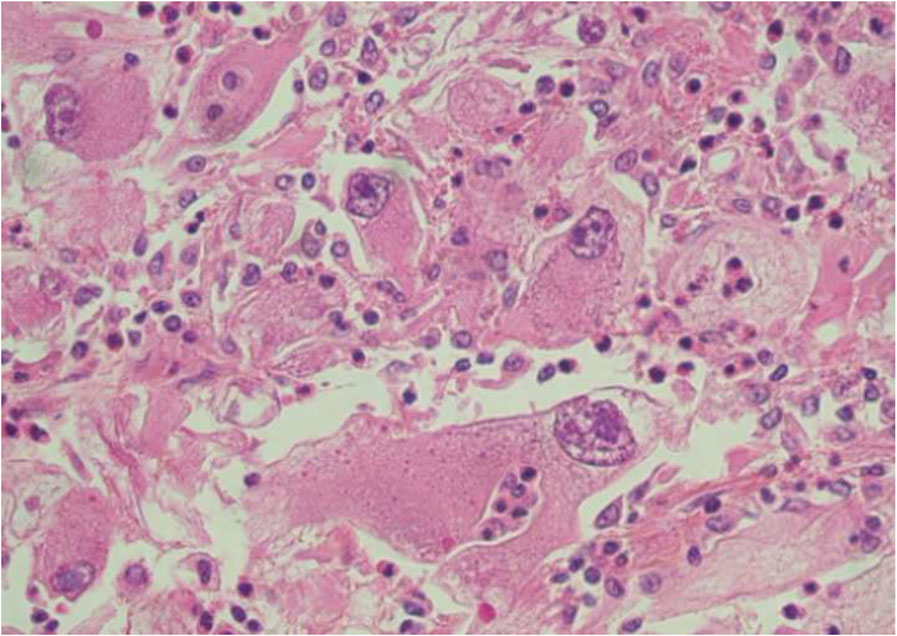
Histological determination of clear cell carcinoma

**Fig. 2:**
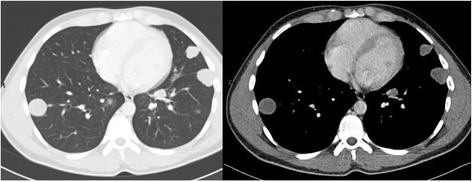
Bilateral lung metastases

**Fig. 3:**
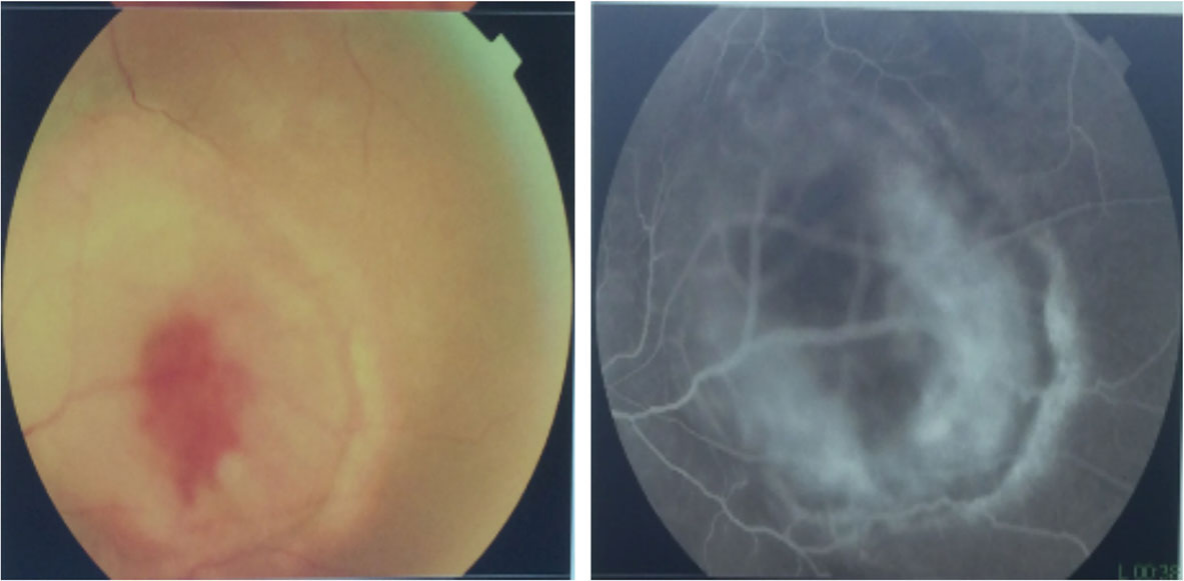
Retinal angiography showing retinal tissue process

**Fig. 4:**
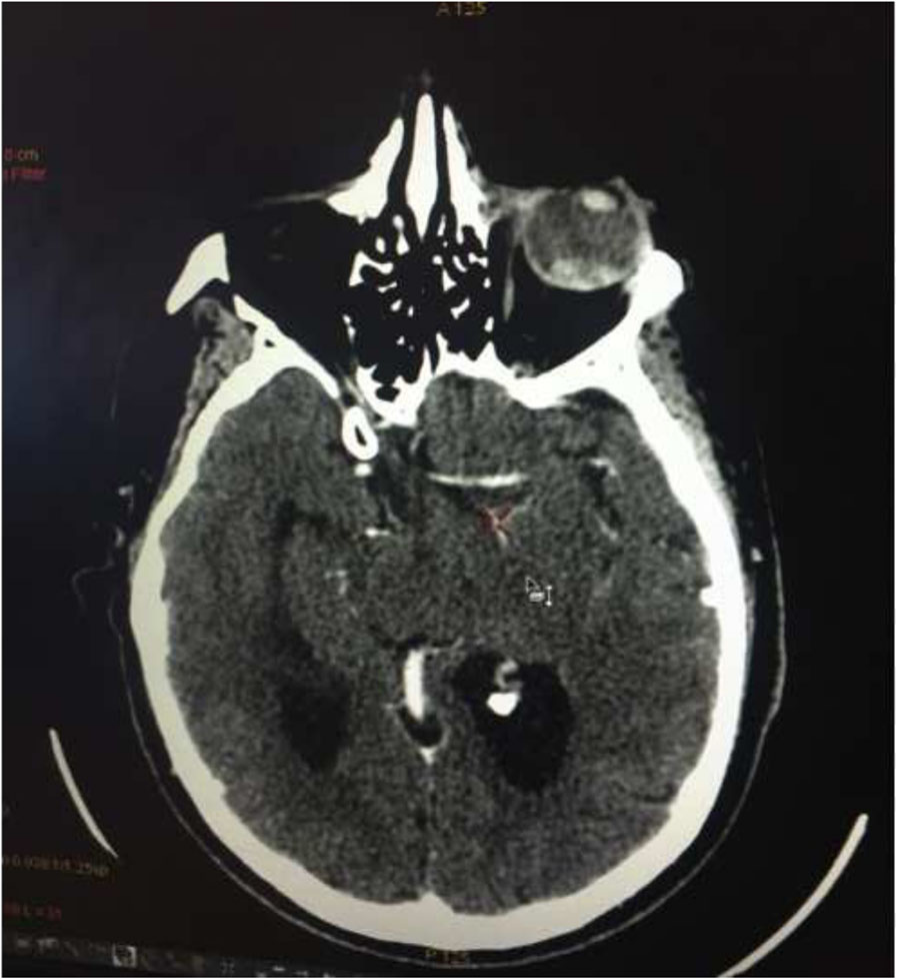
Cross-sectional computed tomographic scan showing a retinal mass

A 62-year-old Moroccan man presented to our hospital with a history of monophthalmitis secondary to a war injury sustained 30 years earlier. He had been followed for 28 months for metastatic clear cell renal carcinoma (Fig. 1). The secondary sites were represented by bilateral pulmonary nodules (Fig. 2). The first-line treatment with sunitinib 50 mg per day (4 weeks on, 2 weeks off) was effective for 24 months and well tolerated. Clinical evaluation demonstrated a large visual field. An ophthalmological assessment with angiography showed the presence of a retinal tissue process (Fig. 3). A cerebral scan confirmed the presence of a retinal metastatic lesion without any cerebral localization (Fig. 4). The rest of the extension report was in favor of an increase in the number and size of pulmonary lesions.

**Fig. 5:**
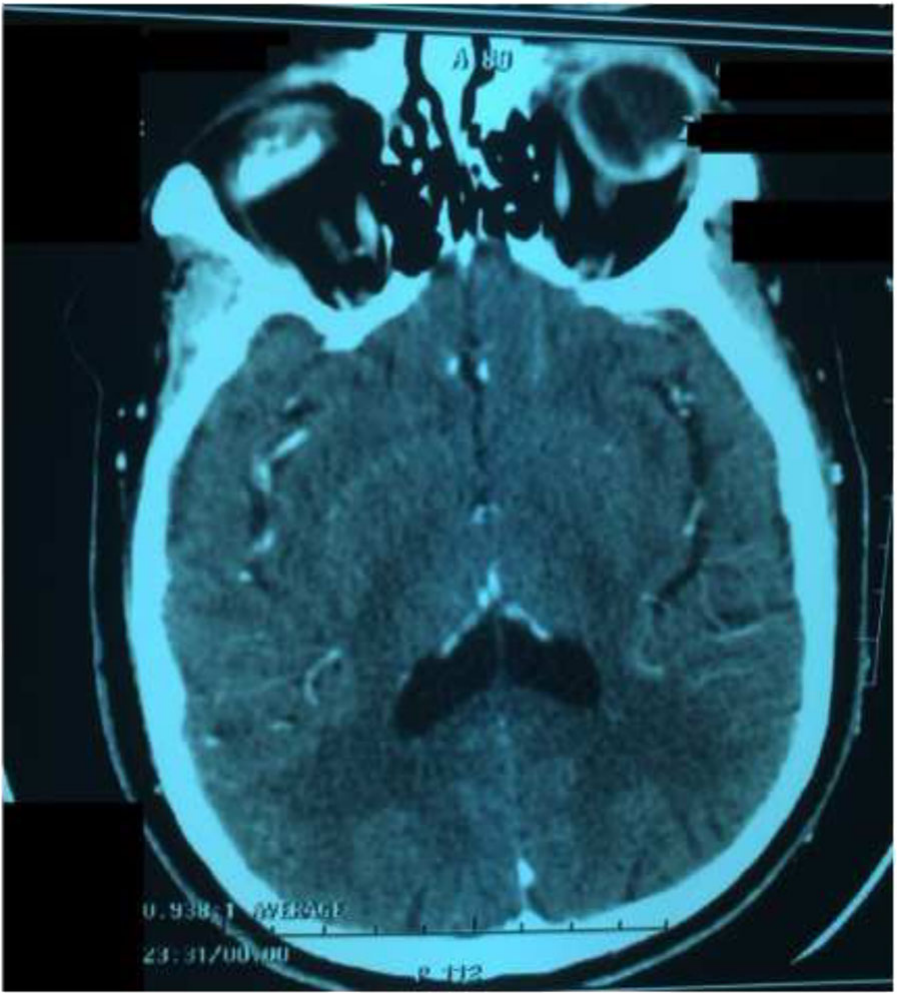
Cross-sectional computed tomographic scan showing responsive retinal mass

A second-line treatment based on everolimus 10 mg daily was started. Evaluation after 4 months revealed marked improvement of symptoms and complete recovery of visual function, despite the persistence of imaging-detected retinal metastasis that was significantly decreased in size (Fig. 5). The use of everolimus was associated with the occurrence of grade 2 mucositis, which rapidly resolved after symptomatic treatment.

## Discussion

Retinal metastases are unusual complications of malignancies [1]. Nevertheless, some authors have reported a recent increase in the incidence of metastases in infrequent sites, such as brain or bone, probably due to the expanded treatment options and the resulting improved survival [7, 8].

The most common intraocular tumor is choroidal metastasis from lung cancer in men and breast cancer in women [8]. In the absence of specific series in the literature, the incidence of renal cancer metastasizing to the retina is certainly even lower than to other sites [9]. The prognosis of choroidal metastasis depends on its primary site [9].

The diagnosis is suspected on the basis of the appearance of visual disturbances and the presence of significant eye pain in a patient followed for cancer [8, 9]. It is confirmed by retinal angiography, which shows nodular detachment [8, 9]. Some imaging methods, such as magnetic resonance imaging (MRI) or computed tomography, can individualize a retinal process [8, 9]. Fine-needle aspiration biopsy may be indicated in some situations, such as uveal metastasis without known primary tumor and patients requesting histopathological confirmation before undergoing recommended therapy such as enucleation [10]. Our patient could not benefit from an MRI, owing to the foreign body persisting within the left orbit since his war injury, which had caused the loss of his left eye 30 years ago. He was followed for evolutionary kidney cancer and did not require histological confirmation. Treatment options include observation if the patient is preterminal or the metastasis appears regressed; systemic therapy (immunotherapy, targeted therapy) or whole-eye radiotherapy if the metastases are multifocal, bilateral, or associated with extensive subretinal fluid; plaque radiotherapy for solitary metastasis; and enucleation, which is considered in very limited cases such as choroidal metastasis that is progressive and causes refractory pain [10–12].

Until recently, metastatic renal cell carcinoma was considered a cancer with a poor prognosis. Treatment options were limited to cytokines (interferon, interleukin 2). Recent years have been marked by the recognition of several small molecules in metastatic renal cancer [13]. Everolimus (RAD001) is an orally administered inhibitor of the mammalian target of rapamycin, a component of an intracellular signaling pathway that regulates cellular metabolism, growth, proliferation, and angiogenesis [13, 14]. Everolimus has become individualized as a serious option after failure of antiangiogenic therapy [14]. Investigators in the RECORD-1 phase III trial compared everolimus with placebo in patients with metastatic renal cell carcinoma that had progressed while they were receiving sunitinib, sorafenib, or both [14]. Median progression-free survival (PFS) was significantly better in the everolimus arm (4.9 vs. 1.9 months; HR 0.33; *P* < 0.001), and 21% of patients had previously received one systemic treatment option. Median overall survival (OS) was similar between the two groups (HR 0.90; 95% CI 0.71–1.14). The most common grade 3/4 adverse events in the everolimus arm were asthenia, stomatitis, pneumonitis, dyspnea, infections, fatigue, dehydration, and abdominal pain [14]. Updated analysis showed median OS of 14.8 months in the everolimus arm vs. 14.4 months in the control group (HR 0.87; *P* = 0.162); however, preplanned crossover occurred in 79.9% of patients and probably confounded the survival benefit. Only 1% of patients in that study achieved a partial objective response [14]. Our patient had a clear partial response estimated to be 50% with a good tolerance of everolimus. Although the benefits of everolimus may need to be evaluated in clinical trials, this may not be feasible, owing to the rarity of the choroidal metastasis of clear cell renal cancer.

Nivolumab is a humanized antibody against programmed cell death protein 1 receptor. Researchers in a pivotal trial (CheckMate 025) enrolled 821 previously treated patients and randomized them to a nivolumab arm (3 mg/kg every 2 weeks) or an everolimus arm (10 mg daily). The primary endpoint of their study was OS. Nivolumab significantly improved median OS (25.0 vs. 19.6 months; HR 0.73; 95% CI 0.60–0.89; *P* = 0.0018). The overall response rate was better with nivolumab (25% vs. 5%), whereas there were no difference in the median PFS (4.6 vs. 4.4 months) [15].

Cabozantinib is a multikinase inhibitor targeting vascular endothelial growth factor receptor, MET, RET, and AXL. These tyrosine kinases are involved in oncogenesis, metastasis, tumor angiogenesis, and drug resistance. METEOR was a phase III study in which researchers randomized 658 previously treated patients to cabozantinib 60 mg daily or to everolimus 10 mg daily. Median PFS (the primary endpoint of the study) was significantly better with cabozantinib (7.4 vs. 3.8 months; HR 0.58; 95% CI 0.45–0.74; *P* < 0.0001). The response rate was also better with cabozantinib (21% vs. 5%) [16]. Both nivolumab and cabozantinib are still not available in Morocco.

## Conclusions

We present a case of a patient followed for metastatic clear cell renal cancer who had a large retinal mass on one eye that appeared while under treatment. This metastatic event is very rare. This case highlights the possibility of successful management of retinal metastasis by medical means. Thus, only a greater awareness of the problem will lead to choosing the best therapeutic approach to individual patients.
